# Assessment of myocardial function in elite athlete’s heart at rest - 2D speckle tracking echocardiography in Korean elite soccer players

**DOI:** 10.1038/srep39772

**Published:** 2016-12-22

**Authors:** Lucy Youngmin Eun, Hyun Wook Chae

**Affiliations:** 1 Division of Pediatric Cardiology, Department of Pediatrics, Yonsei University College of Medicine, Seoul, Korea; 2 Division of Pediatric Endocrinology, Department of Pediatrics, Yonsei University College of Medicine, Seoul, Korea

## Abstract

The purpose of this study was to investigate Korean elite soccer players’ myocardial function using the conventional and advanced speckle tracking imaging to compare the difference with the normal controls. We used 2D echocardiography speckle tracking echocardiography (STE) to evaluate LV regional strain in 29 elite soccer players compared to 29 age-matched healthy controls. Conventional, tissue Doppler, and STI echocardiography was performed, for strain at base and apex, rotation and torsion. There is no difference in longitudinal strain (−17.6 ± 1.8 vs −17.3 ± 2.9, p = ns), and basal radial strain. However, the significant increases were noticed in basal circumferential strain (−17.5 ± 2.6 vs −15.5 ± 8.9, p = 0.05), apical radial strain (33.1 ± 20.5 vs 22.5 ± 19.4, p = 0.02), and apical circumferential strain in soccer players (−21.4 ± 4.8 vs −16.8 ± 7.6, p = 0.005). Soccer players showed the higher rotation at base (−3.9 ± 1.9 vs −2.6 ± 3.2, p = 0.03), and apex (6.98 ± 2.62 vs 6.21 ± 3.81, p = 0.05), higher torsion (10.9 ± 3.7 vs 8.8 ± 6.3, p = 0.05). In conclusion, the elite soccer players’ heart demonstrated the unique ventricular adaptation. These alterations could benefit the cardiovascular adjustment to exercise without much loss of myocardial energy expenditure.

During exercise, the heart endures some alteration as a result of increase heart rate and cardiac output^1^. This is a left ventricular adaptation to long-term intensive strength training characterized by increase in chamber size, wall thickness, and LV mass, as Athlete’s heart^2^,^3^,^4^,^5^.

Athletes show an improvement in myocardial diastolic properties and supernormal left ventricular diastolic function^6^. This can exemplify different morphological heart features in consequences to various kinds of practiced sports.

The previous echocardiographic studies of athlete’s cardiac function have demonstrated that prolonged exercise is concomitant to a shift in diastolic filling from early to late diastole, a change in ventricular dimensions and volumes, a reduction in systolic function, and the development of wall-motion abnormalities in the LV^7^,^8^,^9^.

The tissue Doppler imaging is the most suggested method for quantitative and regional analysis of myocardial function. In addition, recent development of 2D ultrasound speckle tracking echocardiography provided capability of non-invasive evaluation for strain, and assessment of LV torsion.

Investigations of elite athlete’s heart have recently provided relevant information on the apex myocardial reserve of the left ventricle^10^. However, athlete’s heart has not been clarified the myocardial strain alteration at each myofibril direction.

The purpose of this study was to investigate Korean elite soccer players’ myocardial function using the conventional and advanced method with 2D speckle tracking echocardiographic imaging to compare the difference with the healthy normal controls.

## Methods

### Study population

This study included 29 highly trained Korean elite athletes of soccer players, 29 age-matched healthy sedentary controls. The elite soccer players participated in World-cup soccer competition. No one had any past history of cardiovascular disease or related problem or arrhythmia. This study was approved by The Institutional Review Board at Yonsei University. The written informed consent was obtained from each of the participant or their legal guardian for this data publication. All the followed methods were performed in accordance with the relevant guidelines and regulations in Yonsei University.

### Echocardiographic examination

**Figure 1:**
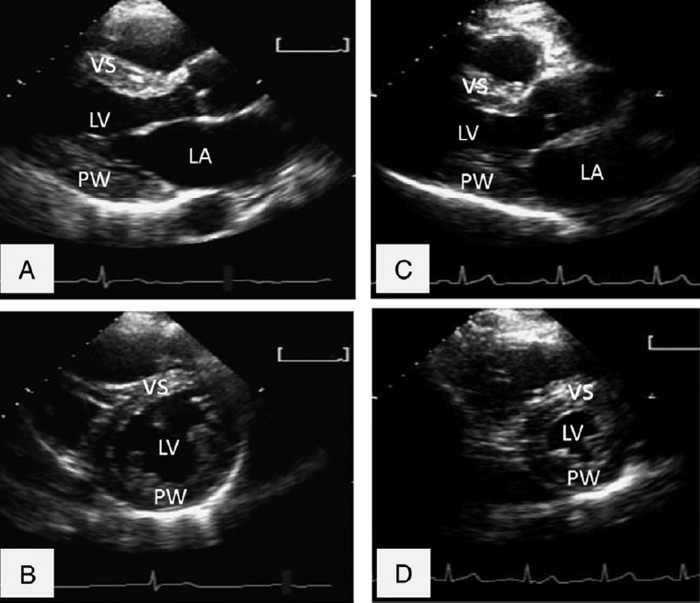
Athlete’s two dimensional echocardiographic images of left ventricle, (**A**) parasternal long axis view, (**B**) parasternal short axis view, comparison with healthy controls’ heart, (**C**) parasternal long axis view, (**D**) parasternal short axis view. VS:ventricular septum. LV:left ventricle. PW: posterior wall.

The quantitative analysis of the left ventricle was performed according to the current recommendations and guideline of the American Society of Echocardiography and the European Association of Echocardiography, using a high quality echocardiography machine (Vivid 7, GE, Milwaukee, WI, USA), equipped with a 2.5 MHz probe. (Fig 1)

LV EF and stroke volume were derived as the average of the measurements in apical four chamber and two chamber views according to the modified Simpson’s rule. LV mass was indexed to height. Relative diastolic wall thickness was determined as twice the posterior wall thickness divided by LV end-diastolic diameter.

Left atrial volume was determined as the average of measurements in apical four chamber and two chamber views and indexed to body surface area.

**Figure 2:**
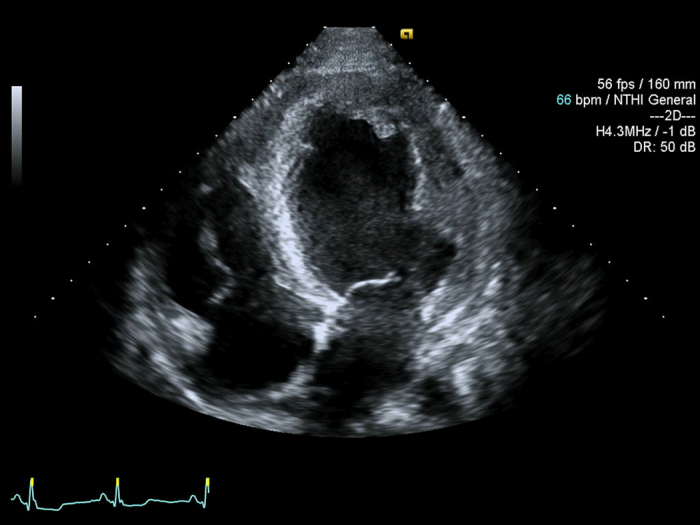
Athlete’s two dimensional echocardiographic image of left ventricle at apical 4 chamber view.

**Figure 3:**
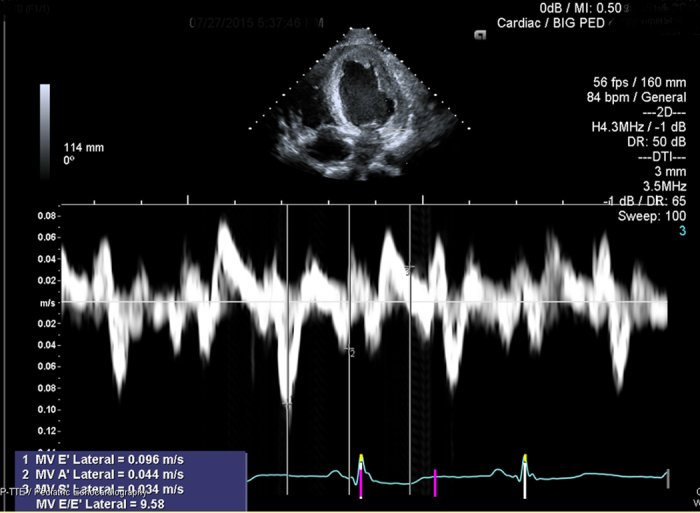
Tissue Doppler velocity at lateral mitral annulus of left ventricle.

Transmitral pulsed wave Doppler and tissue Doppler velocities were recorded in the apical four chamber view at septal and lateral annuli. (Figs 2 and 3)

Speckle tissue tracking was performed according to validated methods on three consecutive cardiac cycles of two dimensional LV images in apical and parasternal view.

2D strain variables were measured in the parasternal short axis and apical four chamber views.

### Speckle tracking echocardiography

2D echocardiographic images at apical 4- and 2- chamber views were obtained using conventional gray scale, during breath hold with a stable ECG recording. The three consecutive heart cycles were recorded and averaged.

Advanced tissue speckle tracking echocardiographic images were recorded at a rate over 50 frames /sec to investigate the myocardial strain and its rate. Two-dimensional images of apical 4 chamber view were recorded for longitudinal strain, while those of parasternal short axis view were acquired for radial and circumferential strain. Offline speckle tracking process was conducted with acoustic markers using dedicated software. The endocardial and epicardial borders were traced manually, the middle-myocardial border automatically defined as the midline between the endocardial border and epicardial border. These three contours were tracked frame by frame throughout the cardiac cycle.

### Torsion

LV torsion was assessed by calculation. The rotational movement as the apex moves with respect to the base about the LV long axis, by speckle tracking.

Averaged LV rotation and rotational velocities from the base and apex were used for calculation of LV torsion.

### Statistics

Data are presented as mean + SD. Paired student *t* test was performed to compare the parameters. Statistical significance was inferred by a *p* value less than 0.05, using Statistical Package for the Social Sciences (SPSS) for Windows software version 20.0 (SPSS Inc, Chicago, IL, USA).

## Results

The soccer players exhibited increased height, weight, body surface area, and lower hear rate. The systolic and diastolic blood pressures were not dissimilar. (Table 1) LV mass and mass index were much increased in soccer players with higher end-systolic wall stress. (Table 2)

LV volume at systole and diastole, and LA volume in systole and diastole, (Table 3) RV systolic area and RA area in systole and diastole were all increased in soccer players. (Table 4) None of the subjects had significant valvular and subvalvular obstruction or a moderate or severe valve insufficiency.

With conventional trans-mitral Doppler parameters, the early diastolic velocity of E was not statistically different, however, the late diastolic velocity of A was decreased, the ratio of E/A was increased, deceleration time was increased in soccer players, pressure half time was not dissimilar (Table 5).

As a marker of advanced diastolic function with TDI, the septal early diastolic E′ was not different, however, septal late diastolic A′, septal systolic S′ were decreased, the ratio of conventional early diastolic flow velocity per septal myocardial early diastolic tissue Doppler velocity of E/E′ was decreased in athletes. Lateral early diastolic E′ was increased, lateral late diastolic A′, lateral systolic S′ were decreased, and the ratio of lateral E/E′ was significantly decreased in athletes (Table 6).

With advanced 2D speckle tracking imaging, myocardial strain analysis was performed. There is no difference in longitudinal strain (−17.6 ± 1.8 vs −17.3 ± 2.9, p = ns), and basal radial strain (Table 1). However, the significant increases were noticed in basal circumferential strain (−17.5 ± 2.6 vs −15.5 ± 8.9, p = 0.05), apical radial strain (33.1 ± 20.5 vs 22.5 ± 19.4, p = 0.02), and apical circumferential strain in soccer players (−21.4 ± 4.8 vs −16.8 ± 7.6, p = 0.005) (Table 7).

Moreover, soccer players showed the higher rotation at base (−3.9 ± 1.9 vs −2.6 ± 3.2, p = 0.03), and apex (6.98 ± 2.62 vs 6.21 ± 3.81, p = 0.05) (Table 8). The calculated torsion from the rotation was also increased in soccer players (10.9 ± 3.7 vs 8.8 ± 6.3, p = 0.05)

## Discussion

A supernormal cardiac function in athlete’s heart, especially about LV diastolic pattern, has been described^11^,^12^,^13^. A complex physiological phenomenon and benign adaptation to exercise conditioning, involved not only cardiac chambers, but also the myofibril structure and function of ventricle^6^,^14^.

This study demonstrated several significant differences between elite soccer players and healthy controls. Mitral inflow Doppler A wave was lower in soccer players with decreased tissue Doppler A′, which explained a shift in the pattern of the diastolic filling from late to early diastole, and this shift may reflect in a fast submissive atrial emptying. The increased measurement of tissue Doppler E′, S′, and decreased measurement of E/E′ in elite soccer players, supported the previous researches about supernormal diastolic function of athletes as well^6^.

The contraction of myocardium induces a systolic increase in LV normal strains, longitudinal shortening, radial thinning, and circumferential shortening. Moreover, due to helical orientation of myofibrils, contraction also induces shear strains within the myocardium, including longitudinal-radial, circumferential-radial, and circumferential-longitudinal elements^11^. In this study, rotation and torsion data were investigated for the concept of helical twist. With the speckle tracking imaging, the LV strains, rotation and torsion, could be recognized to perceive the potential mechanism of the cardiac functional alteration with exercise.

For our result of elite soccer players, the longitudinal strains during systole were similar to controls. At the base, circumferential strain was increased. At the apex, both of radial and circumferential strains were all significantly increased. This result suggested the LV strain of circumferential myofibril is the most sensitive, then, apical circumferential and radial orientation is much delicate than the base. In addition, this result supported the previous researches that the enhanced diastolic alteration during exercise is because of the more dynamic untwisting motion of the apex in LV level^14^,^15^.

The rotation is also significantly increased in athletes at the base and apex in this study.

Torsion calculated from apical and basal rotation is higher in soccer players. LV torsion depends on the LV wall thickness to LV radius ratio, and afterload. Notomi *et al*. insisted that the magnitude of increase in twisting and untwisting rates during exercise was significantly greater than the corresponding changes in LV length and radius^15^. Neilan *et al*. reported that LV torsion increased at post-completion of the race with indoor rowing players^7^. In this study, the elite soccer players showed higher torsion at resting state than healthy controls.

Although, longterm precise measurement is yet limited, the 2D-derived LV torsion is a feasible, sensitive and specific parameter of myocardial function assessment in elite soccer player for athlete’s heart.

## Conclusion

The elite soccer players’ heart demonstrated the unique ventricular adaptation including higher basal circumferential strain, apical radial and circumferential strain, higher basal rotation and apical rotation, and torsion. These alterations could benefit the cardiovascular adjustment to exercise without much loss of myocardial energy expenditure.

## Additional Information

**How to cite this article**: Eun, L. Y. and Chae, H. W. Assessment of myocardial function in elite athlete's heart at rest - 2D speckle tracking echocardiography in Korean elite soccer players. *Sci. Rep.* **6**, 39772; doi: 10.1038/srep39772 (2016).

**Publisher's note:** Springer Nature remains neutral with regard to jurisdictional claims in published maps and institutional affiliations.

## Figures and Tables

**Table 1:** Baseline demographic characteristics of study populations.

Variables	Elite Soccer Players	Healthy Control	*p* values
Age (year)	27.1 ± 4.1	26.9 ± 6.5	ns
Sex (% of male)	29 (100.0)	29 (100.0)	ns
Weight (kg)	76.7 ± 6.0	72.8 ± 9.8	0.05
Height (cm)	182.5 ± 3.5	173.5 ± 4.5	0.001
Body surface area (m^2^)	1.97 ± 0.1	1.87 ± 0.2	0.04
Heart Rate (beats/min)	54.2 ± 10.1	73.3 ± 11.7	0.005
Systolic BP (mmHg)	121.8 ± 8.6	121.1 ± 13.5	ns
Diastolic BP (mmHg)	69.1 ± 10.1	67.3 ± 6.5	ns

BP: blood pressure ns: non-significant.

**Table 2:** Conventional echocardiographic data of left ventricular morphology and function.

Variables	Soccer Players	Control	*p* values
Interventricular septal thickness (mm)	10.4 ± 0.8	9.4 ± 0.9	0.01
Posterior wall thickness (mm)	10.6 ± 0.7	9.3 ± 1.0	0.001
End-diastolic diameter (mm)	53.1 ± 0.7	48.6 ± 3.0	0.001
End-systolic diameter (mm)	36.5 ± 1.8	31.8 ± 2.9	0.001
Relative wall thickness	4.0 ± 0.3	3.9 ± 0.4	ns
LV Mass (g)	216.7 ± 21.9	160.9 ± 31.9	0.001
Mass index (g/height^2^)	110.2 ± 9.4	87.9 ± 14.9	0.001
End-diastolic volume (mL/m^2^)	152.0 ± 19.4	102.8 ± 26.2	0.001
End-systolic volume (mL/m^2^)	62.7 ± 12.7	37.5 ± 15.1	0.001
Stroke volume (mL)	89.3 ± 15.4	65.3 ± 20.8	0.01
Ejection Fraction (%)	62.3 ± 4.2	64.6 ± 5.7	ns
Cardiac index (L/m^2^)	2.5 ± 0.6	2.7 ± 1.0	ns
End-systolic WS (mmHg)	240.9 ± 32.6	149.0 ± 20.9	0.001
End-systolic MWS (g/cm^2^)	21.5 ± 1.5	21.5 ± 2.4	ns

BP: blood pressure, WS: wall stress, MWS: meridional wall stress.

**Table 3:** Left ventricular and left atrium volume data comparison.

Volume (mL)	Soccer Player	Healthy Control	*p* values
LV Systolic Volume (4 chamber)	79.3 ± 17.3	51.9 ± 16.9	0.001
LV Diastolic volume (4 chamber)	219.3 ± 67.3	129.9 ± 36.9	0.001
LV Systolic Volume (2 chamber)	80.6 ± 29.9	46.3 ± 13.9	0.001
LV Diastolic volume (2 chamber)	216.9 ± 71.6	138.4 ± 38.3	0.001
LA Systolic Volume (4 chamber)	67.7 ± 17.3	40.4 ± 13.5	0.001
LA Systolic volume (2 chamber)	86.5 ± 22.1	45.7 ± 20.1	0.001

**Table 4:** Right ventricular and right atrial area data comparison.

	Elite Soccer Player	Healthy Control	*p* values
RV Systolic Area (m^2^)	14.9 ± 3.7	12.2 ± 2.9	0.005
RV Diastolic Area (m^2^)	26.1 ± 7.1	20.1 ± 4.7	0.002
RA Systolic Area (m^2^)	22.2 ± 3.9	15.9 ± 4.1	0.001

**Table 5:** Transmitral inflow Doppler velocity profiles.

	Elite Soccer Player	Healthy Control	*p* values
E-wave (cm/s)	76.6 ± 15.2	81.5 ± 13.8	ns
A-wave (cm/s)	37.4 ± 8.0	52.4 ± 12.7	0.0001
E/A	2.11 ± 0.40	1.79 ± 0.60	0.002
DT (msec)	188.2 ± 19.8	177.7 ± 21.8	0.05
PHT (msec)	56.7 ± 5.5	57.6 ± 3.9	ns

E/A: ration of peak early to peak late transmitral flow velocity, PHT: pressure half time, DT: Deceleration time.

**Table 6:** Tissue Doppler velocities at ventricular septal and mitral lateral annuli.

	Elite Soccer player	Healthy Control	*p* values
Septal E′ (cm/s)	13.0 ± 2.5	12.5 ± 2.2	ns
Septal A′ (cm/s)	7.0 ± 1.5	8.9 ± 1.8	0.001
Septal S′ (cm/s)	8.2 ± 1.2	9.3 ± 1.8	0.01
Septal E/E′	5.96 ± 1.29	6.69 ± 1.05	0.02
Lateral E′ (cm/s)	18.4 ± 3.1	16.0 ± 2.7	0.005
Lateral A′ (cm/s)	6.5 ± 1.4	9.0 ± 2.1	< 0.001
Lateral S′ (cm/s)	11.3 ± 2.0	12.0 ± 2.2	0.01
Lateral E/E′	4.23 ± 0.96	5.21 ± 1.12	0.01

**Table 7:** Radial and Circumferential Strain Comparison between Athletes and Controls.

	Elite Soccer Player	Healthy Control	*P* value
Longitudinal strain	−17.6 + 1.8	−17.3 + 2.9	ns
Base Rad* Strain	40.8 ± 10.7	39.9 ± 23.7	ns
Base Circ# Strain	−17.5 ± 2.6	−15.5 ± 8.9	0.05
Apex Rad* Strain	33.1 ± 20.5	22.5 ± 19.4	0.02
Apex Circ# Strain	−21.4 ± 4.8	−16.8 ± 7.6	0.005

Rad*: radial, Circ#: circumferential.

**Table 8:** Rotation and Torsion Measurements by 2D Speckle Tracking Imaging.

	Elite Soccer Player	Healthy Control	*P* value
Base Rotation	−3.91 ± 1.89	−2.61 ± 3.19	0.03
Apex Rotation	6.98 ± 2.62	6.21 ± 3.81	0.05
Torsion	10.89 ± 3.7	8.82 ± 6.3	0.05
